# Long‐term outcomes of a cascading salvage strategy for high‐risk non‐muscle‐invasive bladder cancer

**DOI:** 10.1111/bju.70044

**Published:** 2025-10-22

**Authors:** Ian M. McElree, Ryan L. Steinberg, Helen Y. Hougen, Sarah L. Mott, Vignesh T. Packiam, Michael A. O'Donnell

**Affiliations:** ^1^ Department of Urology Vanderbilt University Medical Center Nashville TN USA; ^2^ Department of Urology University of Iowa Iowa City IA USA; ^3^ Holden Comprehensive Cancer Center University of Iowa Iowa City IA USA; ^4^ Department of Surgery, Division of Urology Rutgers Cancer Institute of New Jersey New Brunswick NJ USA

**Keywords:** urinary bladder, urinary bladder neoplasms, chemotherapy, clinical protocols, BCG

## Abstract

**Objective:**

To report long‐term oncological outcomes of patients with high‐risk non‐muscle‐invasive bladder cancer (HR‐NMIBC) undergoing bladder‐sparing therapy (BST) after bacillus Calmette–Guérin (BCG) failure.

**Patients and Methods:**

We retrospectively reviewed patients with HR‐NMIBC treated from 2000 to 2025 with intravesical therapies after BCG failure, all of whom received salvage gemcitabine/docetaxel (Gem/Doce). Subsequent BST included valrubicin/docetaxel, sequential doxorubicin/gemcitabine alternating with mitomycin‐C/docetaxel, and gemcitabine/cabazitaxel with pembrolizumab. Kaplan–Meier analysis estimated recurrence‐free survival (RFS), progression‐free survival (PFS), cystectomy‐free survival (CFS), and cancer‐specific survival (CSS).

**Results:**

A total of 85 patients with HR‐NMIBC were included of whom 38 (45%) were BCG‐unresponsive. The median (interquartile range) follow‐up was 86 (43–108) months from induction Gem/Doce. Following Gem/Doce initiation, RFS did not significantly decline with each successive line of salvage therapy: among patients treated with first (Gem/Doce), second, and third line salvage therapy, the 2‐year RFS was 47%, 43%, and 39%, respectively (*P* = 0.68). Overall, the PFS at 5 years was 81% (95% confidence interval [CI] 69–88%). PFS did not significantly decline with each successive salvage line of therapy: among patients treated with first (Gem/Doce), second, and third line salvage therapy, the 2‐year PFS was 91%, 88%, and 93%, respectively (*P* = 0.98). CFS and CSS were 74% (95% CI 62–83%) and 88% (95% CI 79–94%) at 5 years, respectively.

**Conclusions:**

Multi‐line BST is feasible and can yield durable bladder preservation and oncological control in select patients with HR‐NMIBC following BCG failure. Progression‐free outcomes remained favourable across successive lines of salvage therapy. These findings support the role of longitudinal BST as an alternative to radical cystectomy in carefully and appropriately selected patients.

**Abbreviations**
BST: bladder‐sparing therapy
CFS: cystectomy‐free survival
CIS: carcinoma *in situ*
CSS: cancer‐specific survival
GCP: gemcitabine and cabazitaxel with intravenous pembrolizumab
Gem/Doce: gemcitabine and docetaxel
HG: high grade
IFN‐α: interferon alfa
IQR: interquartile range
MFS: metastasis‐free survival
(HR‐)NMIBC: (high‐risk) non‐muscle‐invasive bladder cancer
OS: overall survival
PFS: progression‐free survival
PUC: prostatic urothelial carcinoma
Quad Chemo: sequential doxorubicin and gemcitabine alternating weekly with mitomycin‐C and docetaxel
RC: radical cystectomy
RFS: recurrence‐free survival
UTUC: upper tract urothelial carcinoma
Val/Doce: valrubicin and docetaxel

## Introduction

Non‐muscle‐invasive bladder cancer (NMIBC) comprises the majority of newly diagnosed bladder cancers [1, 2]. Following tumour resection, BCG remains the ‘gold standard’ first‐line therapy for high‐risk NMIBC (HR‐NMIBC). However, a substantial proportion of patients experience recurrence following BCG. While treatment guidelines have recommended radical cystectomy (RC) in this setting, there has been an explosion of approved and emerging novel bladder‐sparing therapy (BST) options [2, 3, 4, 5, 6]. However, long‐term outcomes following these therapies is lacking and there are almost no data regarding efficacy of second and third line salvage therapies. Furthermore, it is unknown how long it is safe to continue BST after NMIBC recurrences. Although curative, RC remains unsuitable or undesired for some patients, and thus, information regarding safety of prolonged BST is valuable [6, 7].

Our institution has explored multiple salvage therapies, primarily multi‐agent sequential intravesical chemotherapeutics, including gemcitabine and docetaxel (Gem/Doce) [8], valrubicin and docetaxel (Val/Doce) [9], sequential doxorubicin and gemcitabine alternating weekly with mitomycin‐C and docetaxel (Quad Chemo) [10], and gemcitabine and cabazitaxel with intravenous pembrolizumab (GCP) [11]. We have developed and utilised a general NMIBC pathway for patients with recurrent NMIBC after BCG who desire bladder preservation. This pathway emphasises advanced surveillance and sequenced salvage therapies, generally starting with Gem/Doce and then progressing to more advanced treatments. Our institutional experience offers a unique dataset depicting the feasibility, efficacy, and safety of a stepwise bladder‐preserving protocol. In the present study, we report long‐term oncological outcomes of patients with HR‐NMIBC undergoing BST after BCG failure at the University of Iowa.

## Patients and Methods

### Study Design and Population

After obtaining Institutional Review Board approval, a retrospective review was conducted of all patients with HR‐NMIBC treated at the University of Iowa between 2000 and 2025. Patients who received Gem/Doce following BCG failure were identified for inclusion if they initiated therapy with the intent to complete a full 6‐week induction course. Risk status was as defined by AUA criteria [2]. Patients were excluded if they did not undergo follow‐up surveillance or if treatment records were incomplete.

### The Iowa NMIBC Treatment Algorithm

At the University of Iowa, patients with HR‐NMIBC are managed using a general BST algorithm with modifications according to patient‐specific circumstances. Initial therapy typically consists of intravesical BCG, administered either at our centre or externally. We have previously administered BCG in combination with interferon alfa (IFN‐α), when it was commercially available, as first‐line therapy given the potential benefit of augmented immunostimulation [12, 13]. For patients who experience persistent or recurrent disease following initial BCG‐based therapy, we also have historically offered combined immunotherapy using BCG with granulocyte‐macrophage colony‐stimulating factor (GM‐CSF), IFN‐α, and interleukin 2 (Quad Immuno) based on the hypothesis that enhanced immunogenic stimulation may overcome mechanisms of BCG failure, including immunosenescence [14, 15].

Once a patient demonstrates evidence of BCG failure (either BCG‐exposed or meeting formal United States Food and Drug Administration [FDA]‐defined criteria for BCG‐unresponsive disease), intravesical therapy with Gem/Doce is typically offered as the first‐line salvage treatment. Subsequent recurrences are typically managed in a step‐wise approach, beginning with Val/Doce then progressing to Quad Chemo and GCP [9, 10, 11]. While this is the general algorithm, the exact sequence is left to surgeon discretion. At each stage, definitive management with RC is discussed within a shared decision‐making framework.

### Surveillance

Our institution employs an enhanced operative restaging procedure for patients with HR‐NMIBC [16]. Enhanced operative restaging procedures include white light cystoscopy with bladder urine cytology plus additional restaging components including blue light cystoscopy, bladder fluorescence *in situ* hybridisation, bilateral retrograde pyelograms, bilateral upper tract wash cytologies, mapping bladder biopsies, and prostatic urethral biopsies. Surveillance intervals are performed according to AUA guidelines [2, 16].

### Analysis

Survival probabilities were estimated and plotted using the Kaplan–Meier method. Estimates along with 95% pointwise CIs were reported. For the cumulative incidence of upper tract disease and prostatic urethra involvement, time was calculated from initial NMIBC diagnosis to development of upper tract and prostatic urethral disease, respectively. To mitigate immortal time bias and standardise outcome assessment across salvage therapies, time zero was defined as the initiation of Gem/Doce induction. Recurrence‐free survival (RFS) was defined as time to recurrence—tumour relapse in the bladder or prostatic urethra in males. Progression‐free survival (PFS) was defined as time to progression—the development of T2 or greater disease, lymph node or metastatic disease. Cystectomy‐free survival (CFS) was defined as time to cystectomy. Otherwise, patients were censored at their last urological follow‐up visit. Cancer‐specific survival (CSS) was defined as time to death due to bladder cancer. Patients were censored at death due to other causes or were last known alive. Overall survival (OS) was defined as time to death from any cause. Patients still alive were censored at last known alive. The conditional Cox regression model proposed by Prentice, Williams, and Peterson (PWP) was used to evaluate differences in RFS and PFS between first‐ (Gem/Doce), second‐ and third‐line salvage therapy. A robust sandwich estimator was used to account for potential dependencies within patients.

## Results

### Cohort Characteristics

A total of 85 patients with HR‐NMIBC who initiated BST with Gem/Doce following BCG failure were included (Table 1). The median (interquartile range [IQR]) follow‐up was 86 (43–108) months from the initiation of Gem/Doce. All patients had high‐grade (HG) tumours, with 40% of patients with any history of T1 and 64% with any history of carcinoma *in situ* (CIS) prior to Gem/Doce induction. Prior to initiating BST, all patients were BCG‐exposed and 38 (45%) were categorised as BCG unresponsive. The median (IQR) time from last BCG exposure to Gem/Doce initiation was 10 (5–21) months. At the time of Gem/Doce start, the median (IQR) number of lines of prior treatment was 2 (1–2).

**Table 1:** Cohort characteristics prior to Gem/Doce induction.

Variable	Value
Total number of patients	85
Age at Gem/Doce, years, median (IQR)	73 (67–78)
Gender, *n* (%)	
Female	18 (21)
Male	67 (79)
Pre‐Gem/Doce pathology, *n* (%)	
CIS	31 (36)
T1HG	13 (15)
T1HG + CIS	9 (11)
TaHG	15 (18)
TaHG + CIS	12 (15)
Positive urine cytology	5 (6.1)
History of T1*, *n* (%)	
No	51 (60)
Yes	34 (40)
History of CIS*, *n* (%)	
No	31 (36)
Yes	54 (64)
BCG exposure*, *n* (%)	
BCG unresponsive	38 (45)
BCG exposed	47 (55)

* Prior to Gem/Doce induction.

### Oncological Outcomes

**Fig. 1:**
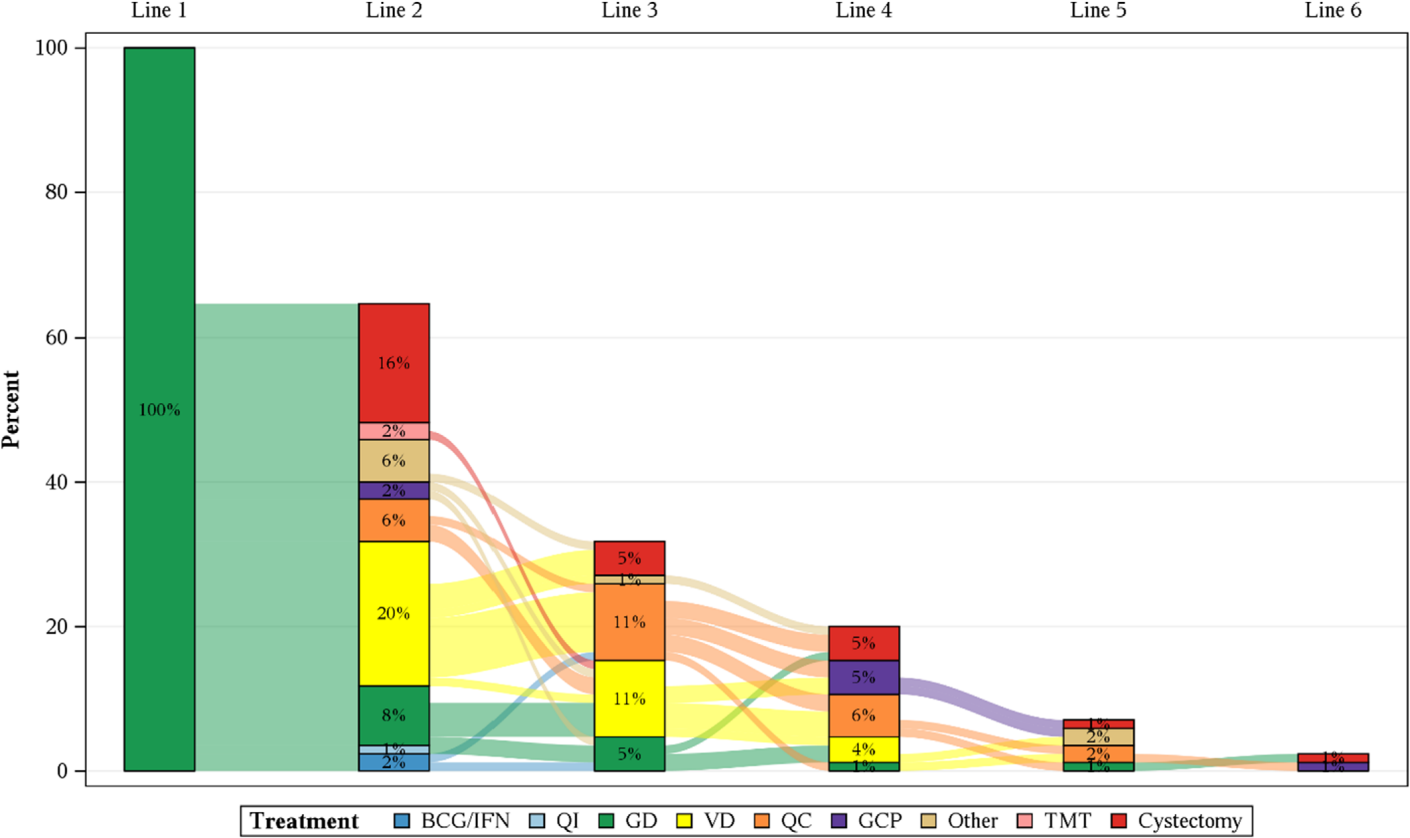
Treatment trajectories among patients with HR‐NMIBC managed with bladder‐sparing intent following initiation of Gem/Doce. ‘Other’ category treatments included docetaxel, adenovirus IFN‐α, nadofaragene firadenovec, radiation, and chemoradiation. GD, Gem/Doce; QC, Quad Chemo; QI, Quad Immuno; TMT, trimodal therapy; VD, Val/Doce.

**Fig. 2:**
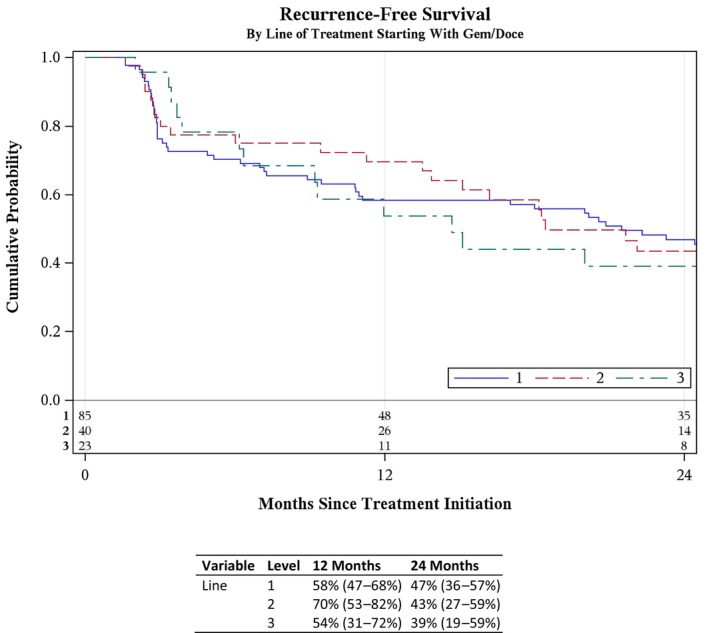
The RFS by line of salvage therapy.

Following Gem/Doce induction, the 1‐ and 2‐year RFS rates were 58% (95% CI 47–68%) and 47% (95% CI 36–57%), respectively. Second‐line salvage treatment included Val/Doce (20%), RC (16%), Gem/Doce (8%), Quad Chemo (6%), other (6%), BCG‐containing regimen (3%), GCP (2%), and systemic therapies (2%). Third‐line salvage therapies included Val/Doce (11%), Quad Chemo (11%), Gem/Doce (5%), RC (5%), and other (1%). Treatment trajectories are shown in Figs 1, S1 and S2. The median (IQR) number of total intravesical therapies received was 3 (3–5). RFS did not significantly decline with each successive line of BST: among patients treated with first‐ (Gem/Doce), second‐, and third‐line salvage therapy, the 2‐year RFS was 47% (95% CI 36–57%), 43% (95% CI 27–59%), and 39% (95% CI 19–59%), respectively (*P* = 0.68; Fig. 2). In the subset of patients with BCG‐unresponsive disease, the 2‐year RFS for first‐ and second‐line salvage therapy was 43% (95% CI 27–58%) and 48% (95% CI 25–68%), respectively. Too few patients received third‐line therapy with sufficient follow‐up to reliably estimate RFS. These preliminary data suggest that recurrence risk did not increase between the first and second line of BST in this contemporary population of greatest clinical relevance.

A table describing oncological outcomes is presented in Table 2. A total of 24 patients ultimately underwent RC (Table S1). CFS was 74% (95% CI 62–83%) at 5 years (Fig. S3). Two patients received RC due to end‐stage bladder symptoms, both after a total of five intravesical treatments. At the time of RC, six patients exhibited disease progression: three with MIBC‐N+, two NMIBC‐N+, and one MIBC‐N0. Among the five patients that were node‐positive at the time of RC, the number of prior treatment lines was seven, six, four, three, and three, respectively. Despite counselling for RC, two patients delayed RC for 14 and 26 months, with pathology showing T1N1 and TisN2, respectively. One patient developed a lesion at the urachal remnant with follow‐up MRI demonstrating perivesical fat invasion and a nonspecific 0.7‐cm node; pathology showed T3aN3. Another developed MIBC after third‐line intravesical therapy; pathology showed T3bN3. One patient developed pan‐urothelial CIS of the bladder, upper tracts, and prostatic urethra and had RC delayed several months due to undergoing surgery at an outside hospital; pathology showed pT3N2 disease.

**Table 2:** Cumulative oncological outcomes.

Outcome, % (95% CI)	1 year	3 years	5 years	7 years
PFS	95 (88–98)	87 (77–93)	81 (69–88)	75 (62–84)
MFS	98 (91–99)	94 (85–97)	89 (78–94)	84 (72–91)
CFS	88 (79–93)	81 (70–88)	74 (62–83)	68 (55–78)
CSS	99 (92–100)	91 (83–96)	88 (79–94)	83 (72–90)
OS	98 (91–99)	82 (72–89)	70 (58–78)	58 (46–68)

Time zero was defined as the initiation of Gem/Doce induction.

A significant proportion of patients developed disease in the upper tracts or urethra. Prior to initiating Gem/Doce, 11 patients exhibited upper tract urothelial carcinoma (UTUC) and eight had prostatic urethral carcinoma (PUC). During follow‐up, 21 patients developed UTUC and nine developed PUC. Of those developing UTUC, 21 received topical therapy and 13 received radical nephroureterectomy. Of those developing PUC, 14 received additional BST and three proceeded directly to RC. At 5 years after initiating Gem/Doce, the cumulative incidence of developing upper tract disease was 27% (95% CI 16–38%), and developing prostatic urethral disease (among those with a prostate) was 18% (95% CI 9–31%).

**Fig. 3:**
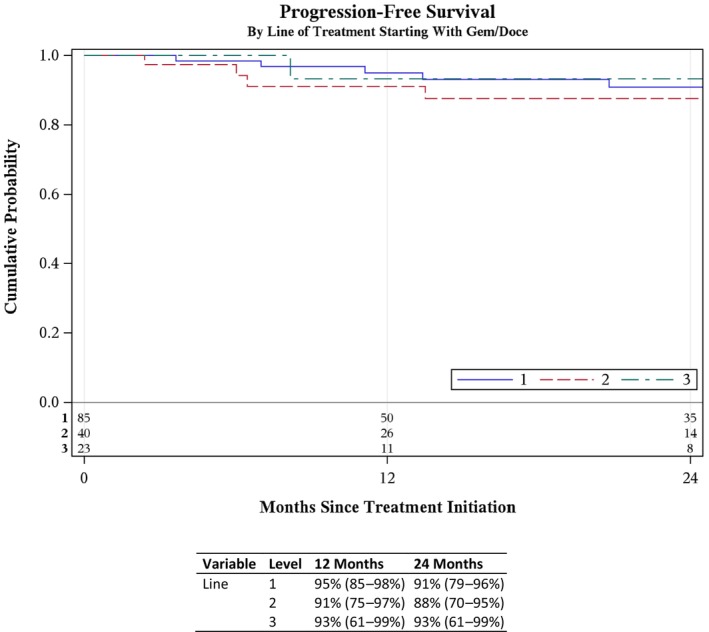
The PFS by line of salvage therapy.

Beyond upper tract or urethral disease, 14 patients experienced metastasis resulting in metastasis‐free survival (MFS) of 89% (95% CI 78–94%) at 5 years (Fig. S4). Cumulative PFS at 5 years was 81% (95% CI 69–88%, *P* = 0.98; Fig. S5). PFS did not significantly decline with each successive line of salvage therapy: among patients treated with first‐ (Gem/Doce), second‐, and third‐line salvage therapy, the 2‐year PFS was 91% (95% CI 79–96%), 88% (95% CI 70–95%), and 93% (95% CI 61–99%), respectively (Fig. 3). At 5‐years post‐Gem/Doce, CSS and OS were 88% (95% CI 79–94%; Fig. S6) and 70% (95% CI 58–78%; Fig. S7), respectively.

When considering oncological outcomes at 1, 2, and 5 years in patients with BCG‐unresponsive NMIBC: RFS was 60%, 43%, and 29% (Fig. S8); PFS was 97%, 91%, and 84% (Fig. S9); CFS was 89%, 84%, and 74% (Fig. S10); MFS was 97%, 94%, and 90% (Fig. S11); CSS was 97%, 95%, and 88% (Fig. S12); and OS was 97%, 92%, and 66% (Fig. S13), respectively. When comparing these outcomes by BCG‐unresponsive vs BCG‐exposed status, oncological outcomes were similar, and all CIs overlapped (Figs S8–S13).

## Discussion

In this real‐world evaluation of patients with HR‐NMIBC following BCG failure, we found that a longitudinal BST programme was feasible, well‐tolerated, and associated with durable oncological outcomes. Extended bladder preservation was achievable for many patients who declined immediate RC with an estimated 74% CFS at 5 years post‐Gem/Doce. While some patients ultimately experienced disease progression, long‐term outcomes remained favourable with an estimated 89% MFS and 88% CSS at 5 years after Gem/Doce induction. When comparing outcomes across successive lines of therapy, neither RFS nor PFS declined significantly with successive treatments, suggesting that continued BST may remain effective for well‐selected patients beyond initial salvage treatment. Notably, in the BCG‐unresponsive subset, 2‐year RFS rates for first‐ and second‐line BST were similar (43% and 48%, respectively), suggesting that recurrence risk across salvage lines may not be as steep as historically assumed. This cohort, drawn from a centre committed to innovative bladder‐sparing strategies, provides the first real‐world long‐term data on the efficacy and safety of multiple successive salvage therapies, and new insight into how far RC can be deferred in carefully selected patients.

The Iowa BST programme was developed in response to limited salvage options, rising patient preference for bladder preservation, and recognition that durable NMIBC remission can be achieved without immediately resorting to RC. During the BCG shortage, salvage Gem/Doce was routinely offered to patients following BCG failure, providing a HG‐RFS rate of 60% at 1 year, and 30% at 5 years [8]. Importantly, PFS was 82% at 5 years post‐induction, suggesting there existed a window of opportunity to safely explore BST [8]. We examined Val/Doce in a population of patients typically failing both BCG and Gem/Doce [9]. At 3‐years, RFS was 41% and PFS was 84% [9]. Patients with recurrence who declined or were ineligible for RC were offered Quad Chemo, which achieved a 2‐year RFS of 43% [10]. Unfortunately, progression events increased over time with a 5‐year PFS of 57% [10]. Most recently, our group developed and began offering GCP, which yielded a 2‐year RFS of 52% and PFS of 70% [11]. Despite concerns about higher progression risk with continued BST, the present study found promising outcomes beyond first‐line therapy, suggesting bladder preservation remains a viable treatment option beyond initial salvage therapy when guided by close surveillance and multidisciplinary care.

Given its potential for definitive cancer control, RC remains a standard of care for patients with recurrent disease after BCG failure [2, 17]. However, our multi‐line BST approach achieved favourable long‐term outcomes, with 89% MFS, 88% CSS, and 74% CFS at 5 years, comparable to those reported for upfront RC. A recent study evaluating outcomes of patients with BCG‐unresponsive NMIBC found no significant differences in MFS, CSS, or OS following RC vs continued BST [18]. In a separate study, patients undergoing immediate RC for HR‐NMIBC demonstrated a 5‐year MFS of 79% [19]. Notably, the early RC cohort in this study had a high‐risk profile: 75% harboured T1 disease and 7.6% demonstrated lymphovascular invasion immediately prior to surgery [19]. Even so, these findings suggest that, in appropriately selected patients, BST can achieve long‐term oncological outcomes similar to those observed with upfront RC.

Bladder preservation may also offer quality‐of‐life advantages by avoiding the physical and psychological burdens of RC and urinary diversion [20]. Although, recent results from the CISTO trial (ClinicalTrials.gov identifier: NCT03933826) have challenged this assumption [7]. In this prospective study of patients with recurrent NMIBC, most quality of life endpoints were similar or better among participants who chose RC vs BST [7]. Notably, PFS was worse in the RC group due to pathological upstaging at time of surgery, but CSS was similar at 1 year [7]. Though lacking long‐term observation, these findings highlight the importance of individualised decision‐making, as complex patient‐specific factors likely influence the relative benefits of BST vs RC. Future research is needed to better define which patients are most likely to benefit from each approach.

The BST programme initiated at the University of Iowa aligns with several of the guiding principles outlined in a recent International Bladder Cancer Group (IBCG) consensus statement for BCG‐unresponsive NMIBC [6]. This statement emphasises a flexible, patient‐tailored treatment approach, incorporating both approved and off‐label agents following BCG failure including Gem/Doce, nadofaragene firadenovec, nogapendekin alfa‐inbakicept, hyperthermic MMC, and pembrolizumab depending upon eligibility and tumour stage. While helpful in guiding initial salvage therapy, the statement provides little guidance regarding treatment sequencing. In contrast, the Iowa BST programme emphasises a longitudinal pathway, supported by real‐world outcomes across multiple lines of treatment. While our protocol emphasises regimens with demonstrated institutional effectiveness, these therapies have not yet been evaluated prospectively. Nevertheless, our findings demonstrate that such an approach can be safely and effectively delivered.

This study centres on salvage Gem/Doce following BCG failure, but Gem/Doce has also performed favourably as a first‐line treatment for HR‐NMIBC. Based on promising results and BCG scarcity, Gem/Doce was adopted as the de facto first‐line treatment option for HR‐NMIBC starting in 2019 at our institution. Often in this setting, BCG is used as salvage following Gem/Doce failure, after which patients proceed through our general BST pathway. In a small series, outcomes from this sequence were similar to BCG followed by salvage Gem/Doce [21]. While these findings suggest Gem/Doce may be a viable first‐line alternative, the ongoing BRIDGE trial (NCT05538663), a randomised comparison of Gem/Doce and BCG, will help clarify its role in initial NMIBC management [22]. Regardless of sequencing, durable bladder preservation is less dependent on the order of therapies than on the principle that pursuing multiple effective treatments in sequence is both feasible and safe.

This study has limitations. First, its retrospective, single‐institution design introduces the potential for selection bias and limits generalisability. Patients who received multiple lines of salvage therapy may represent a more closely monitored subset with favourable disease biology or better access to care. Second, although all patients were BCG‐exposed, approximately half did not meet the strict FDA criteria for BCG‐unresponsive disease, reflecting real‐world practice but complicating comparisons with clinical trials restricted to that population. Many agents used in this algorithm are not FDA approved, making it unclear whether these results are applicable to those more commonly used in practice today. The results presented here come from a high‐volume centre with advanced surveillance protocols [16]. Finally, our BST algorithm, while effective in this cohort, has not been validated in a prospective or multi‐institutional setting. Future studies should aim to prospectively evaluate risk‐adapted, multi‐line BST with standardised toxicity and patient‐reported outcomes.

In conclusion, this cohort with long‐term follow‐up demonstrates that a flexible, multi‐line BST programme can achieve durable oncological outcomes and extended bladder preservation in well‐selected patients with HR‐NMIBC following BCG failure. Continued BST was both feasible and effective when paired with rigorous surveillance and multidisciplinary care. Prospective studies are needed to validate this approach, clarify optimal patient selection, and compare long‐term oncological and quality‐of‐life outcomes to other contemporary therapies.

## Author Contributions

Conceptualisation, Ian M. McElree, Michael A. O'Donnell; Methodology, Michael A. O'Donnell, Vignesh T. Packiam; Software, Ian M. McElree, Sarah L. Mott; Validation, Michael A. O'Donnell, Vignesh T. Packiam; Formal Analysis, Sarah L. Mott; Investigation, Michael A. O'Donnell, Vignesh T. Packiam, Ryan L. Steinberg, Ian M. McElree; Resources, Michael A. O'Donnell, Sarah L. Mott; Data Curation, Ian M. McElree; Writing – Original Draft Preparation, Ian M. McElree, Sarah L. Mott; Writing – Review and Editing, Michael A. O'Donnell, Helen Y. Hougen, Vignesh T. Packiam, Ryan L. Steinberg; Visualisation, Ian M. McElree; Supervision, Michael A. O'Donnell, Vignesh T. Packiam; Project Administration, Michael A. O'Donnell, Vignesh T. Packiam; Funding Acquisition, Michael A. O'Donnell, Sarah L. Mott.

## Disclosure of Interests

Ian M. McElree, Ryan L. Steinberg, Sarah L. Mott, and Helen Y. Hougen have no disclosures. Vignesh T. Packiam has the following consulting disclosures: Valar Labs, Veracyte, Photocure, Urogen, Ferring, Johnson & Johnson. Michael A. O'Donnell has the following consulting disclosures: Abbott, Photocure, Fidia, Merck, Theralase, Urogen.

## Consent

Informed consent was waived due to the retrospective nature of this project.

## Supporting information


**Table S1.** Clinical and pathological features of patients undergoing RC.
**Fig. S1.** Sankey diagram illustrating full treatment trajectories including prior BCG.
**Fig. S2.** Longitudinal treatment and recurrence patterns in patients with HR‐NMIBC managed with sequential intravesical therapy.
**Fig. S3.** The CFS following salvage Gem/Doce.
**Fig. S4.** The MFS following salvage Gem/Doce.
**Fig. S5.** The PFS following salvage Gem/Doce.
**Fig. S6.** The CSS following salvage Gem/Doce.
**Fig. S7.** The OS following salvage Gem/Doce.
**Fig. S8.** The RFS following salvage Gem/Doce among patients with BCG‐unresponsive disease.
**Fig. S9.** The PFS following salvage Gem/Doce among patients with BCG‐unresponsive disease.
**Fig. S10.** The CFS following salvage Gem/Doce among patients with BCG‐unresponsive disease.
**Fig. S11.** The MFS following salvage Gem/Doce among patients with BCG‐unresponsive disease.
**Fig. S12.** The CSS following salvage Gem/Doce among patients with BCG‐unresponsive disease.
**Fig. S13.** The OS following salvage Gem/Doce among patients with BCG‐unresponsive disease.

## Data Availability

Data included in this study is not publicly available.
