# Supplementary material for: Long‐term outcomes of a cascading salvage strategy for high‐risk non‐muscle‐invasive bladder cancer
Source: BJU Int. 2025 Oct 22;137(2):339–47. doi: 10.1111/bju.70044 (PMC12789842; doi:10.1111/bju.70044)
Supplement: Supplementary file 1 — Table S1. Clinical and pathological features of patients undergoing RC. Fig. S1. Sankey diagram illustrating full treatment trajectories including prior BCG. Fig. S2. Longitudinal treatment and recurrence patterns in patients with HR‐NMIBC managed with sequential intravesical therapy. Fig. S3. The CFS following salvage Gem/Doce. Fig. S4. The MFS following salvage Gem/Doce. Fig. S5. The PFS following salvage Gem/Doce. Fig. S6. The CSS following salvage Gem/Doce. Fig. S7. The OS following salvage Gem/Doce. Fig. S8. The RFS following salvage Gem/Doce among patients with BCG‐unresponsive disease. Fig. S9. The PFS following salvage Gem/Doce among patients with BCG‐unresponsive disease. Fig. S10. The CFS following salvage Gem/Doce among patients with BCG‐unresponsive disease. Fig. S11. The MFS following salvage Gem/Doce among patients with BCG‐unresponsive disease. Fig. S12. The CSS following salvage Gem/Doce among patients with BCG‐unresponsive disease. Fig. S13. The OS following salvage Gem/Doce among patients with BCG‐unresponsive disease. [file BJU-137-339-s001.docx]

Supplementary Table 1: Clinical and pathological features of patients undergoing radical cystectomy (RC)

|  |  |  |  |
| --- | --- | --- | --- |
| **ID#** | **RC Pathology** | **Time to RC (months)*** | **RC Indication** |
| 1 | TisN0 | 106 | end-stage bladder |
| 2 | T0N0 | 33 | end-stage bladder |
| 3 | T1TisN0 | 37 | recurrent NMIBC |
| 4 | T0N0 | 5 | recurrent NMIBC |
| 5 | T0N0 | 2 | recurrent NMIBC |
| 6 | TaTisN0 | 5 | recurrent NMIBC |
| 7 | T0N0 | 7 | recurrent NMIBC |
| 8 | TisN0 | 5 | recurrent NMIBC |
| 9 | TisN0 | 22 | recurrent NMIBC |
| 10 | T1N0 | 4 | recurrent NMIBC |
| 11 | T3N2 | 21 | recurrent NMIBC |
| 12 | TisN0 | 6 | recurrent NMIBC |
| 13 | T1TisN0 | 7 | recurrent NMIBC |
| 14 | T0N0 | 45 | recurrent NMIBC |
| 15 | T1N0 | 3 | recurrent NMIBC |
| 16 | TisN0 | 20 | recurrent NMIBC + UTUC |
| 17 | TisN2 | 106 | MIBC |
| 18 | T3aN3 | 76 | MIBC |
| 19 | T1N1 | 36 | MIBC |
| 20 | TisN0 | 75 | MIBC |
| 21 | T3bN3 | 69 | MIBC |
| 22 | TisN0 | 48 | MIBC |
| 23 | T4aN0 | 9 | prostatic stromal invasion |
| 24 | T0N0 | 30 | prostatic stromal invasion |
| * Time from initiation of Gem/Doce to RC. | | | |


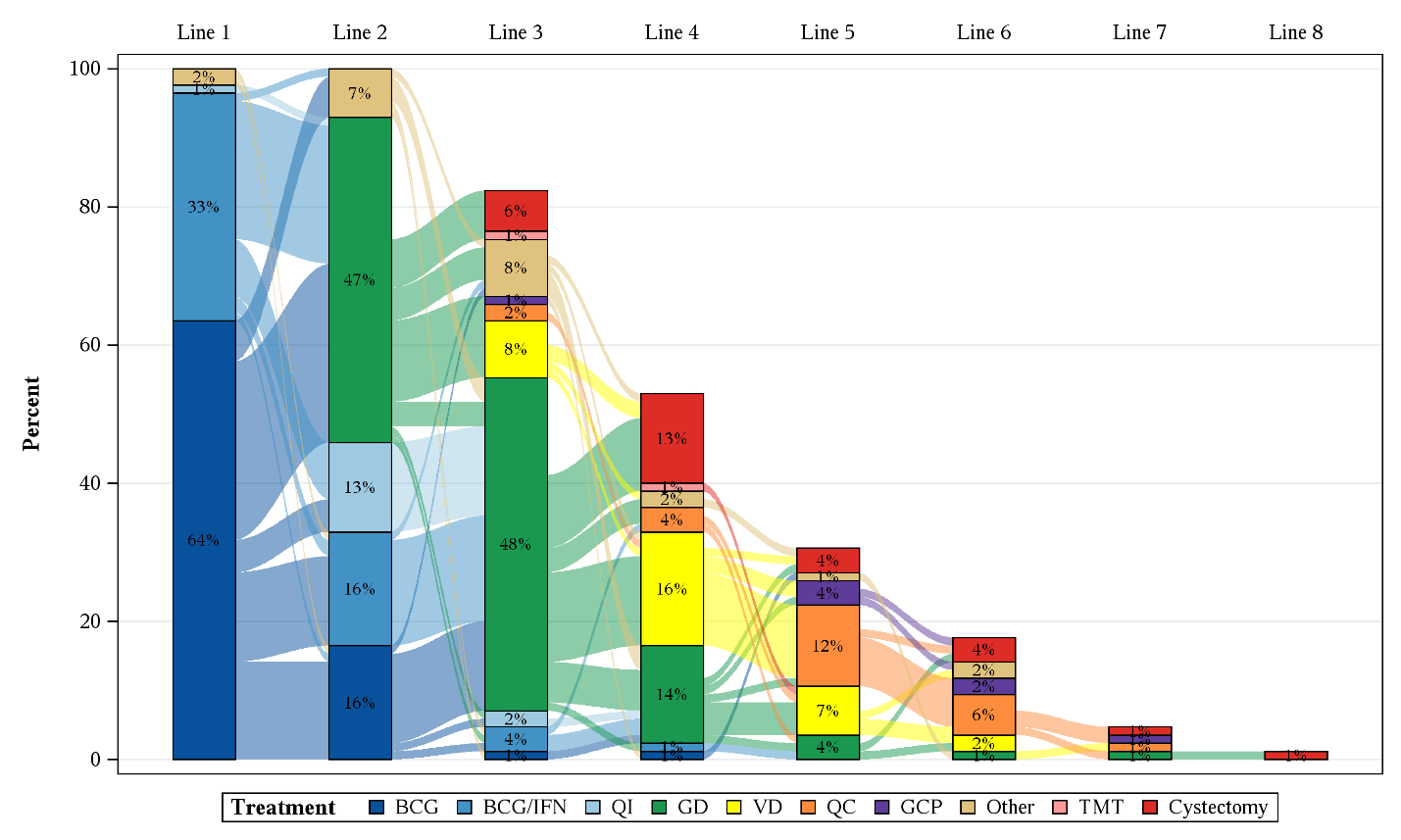
Supplementary Figure 1: Sankey diagram illustrating full treatment trajectories including prior BCG.


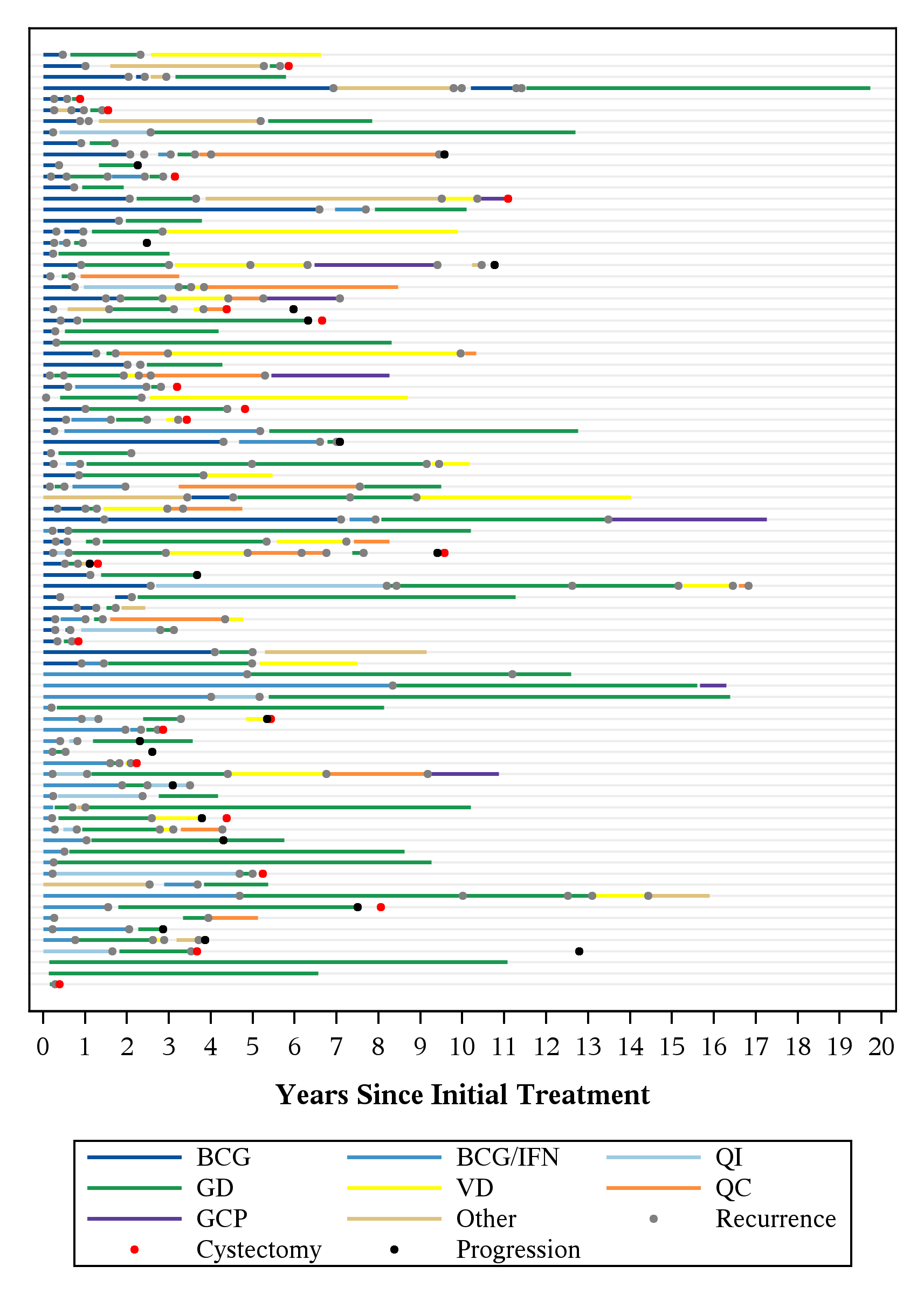
Supplementary Figure 2. Longitudinal treatment and recurrence patterns in patients with HR-NMIBC managed with sequential intravesical therapy.

Supplementary Figure 3: Cystectomy-free survival following salvage Gem/Doce

| **12 Months** | **24 Months** | **36 Months** | **48 Months** | **60 Months** | **72 Months** | **84 Months** | **96 Months** | **108 Months** | **120 Months** |
| --- | --- | --- | --- | --- | --- | --- | --- | --- | --- |
| 88% (79-93%) | 84% (74-90%) | 81% (70-88%) | 76% (64-84%) | 74% (62-83%) | 72% (60-81%) | 68% (55-78%) | 68% (55-78%) | 60% (44-73%) | 60% (44-73%) |


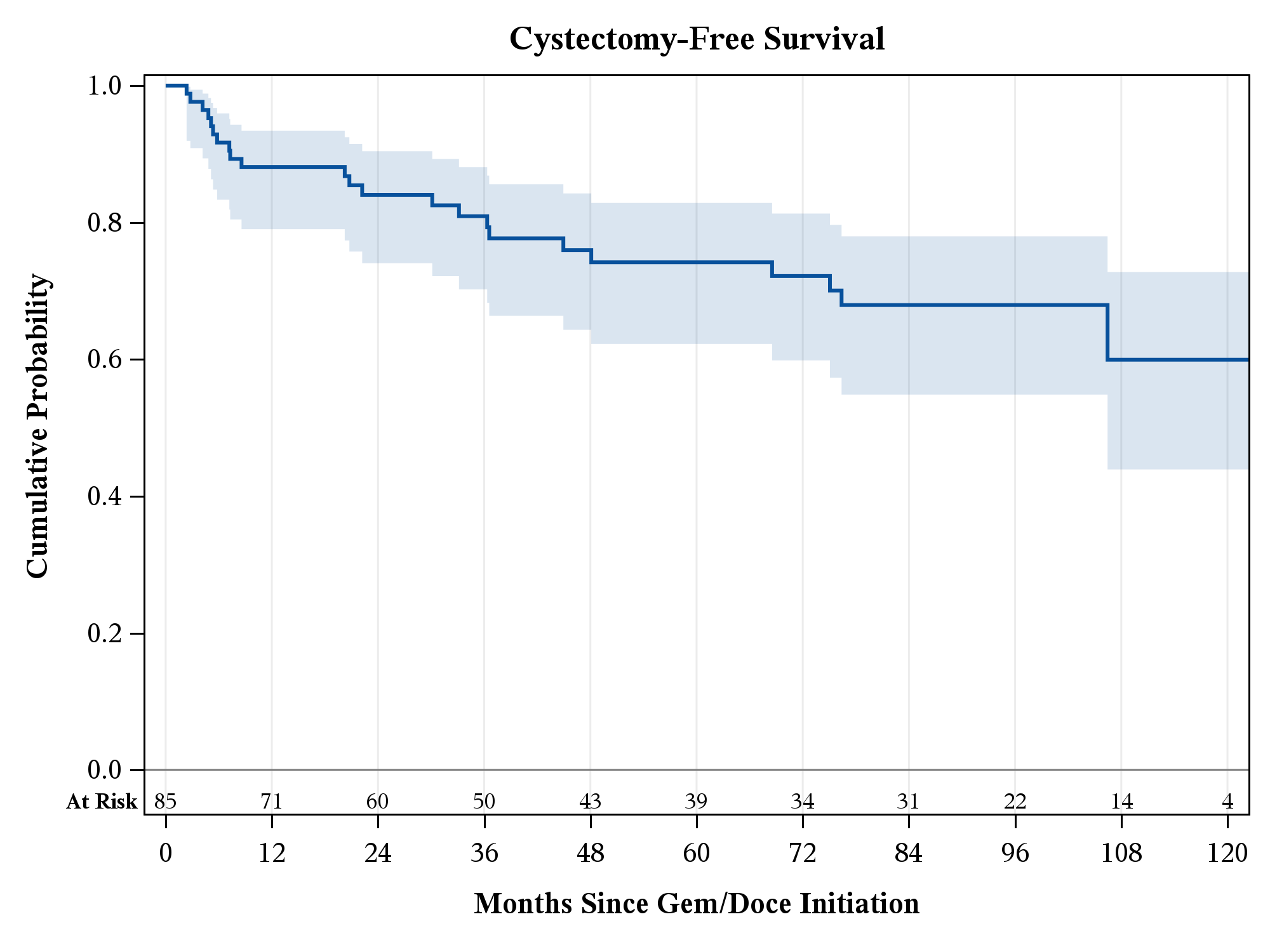


Supplementary Figure 4: Metastasis-free survival following salvage Gem/Doce


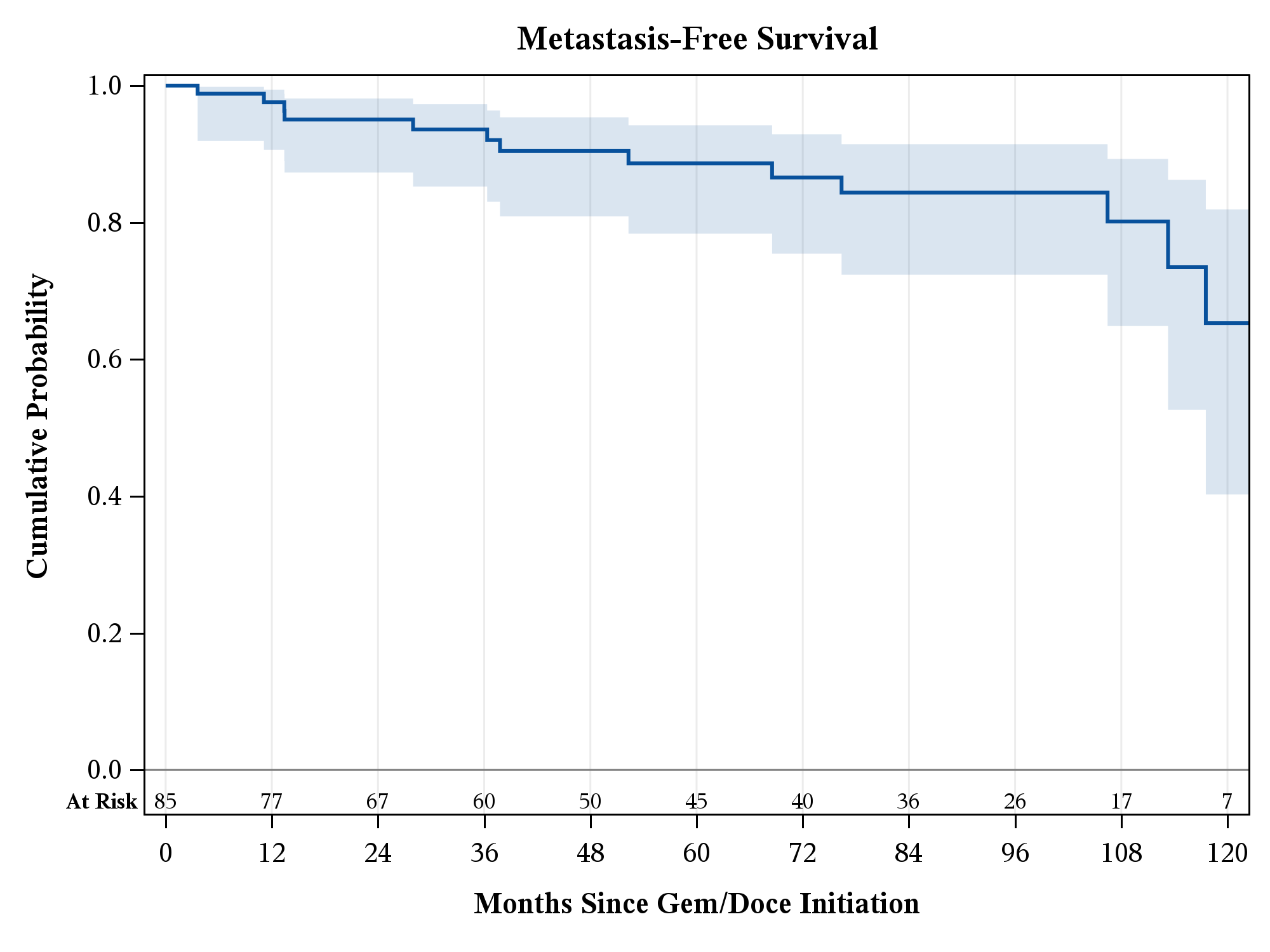


| **12 Months** | **24 Months** | **36 Months** | **48 Months** | **60 Months** | **72 Months** | **84 Months** | **96 Months** | **108 Months** | **120 Months** |
| --- | --- | --- | --- | --- | --- | --- | --- | --- | --- |
| 98% (91-99%) | 95% (87-98%) | 94% (85-97%) | 90% (81-95%) | 89% (78-94%) | 87% (75-93%) | 84% (72-91%) | 84% (72-91%) | 80% (65-89%) | 65% (40-82%) |

Supplementary Figure 5: Progression-free survival following salvage Gem/Doce

| **12 Months** | **24 Months** | **36 Months** | **48 Months** | **60 Months** | **72 Months** | **84 Months** | **96 Months** | **108 Months** | **120 Months** |
| --- | --- | --- | --- | --- | --- | --- | --- | --- | --- |
| 95% (88-98%) | 91% (83-96%) | 87% (77-93%) | 82% (71-89%) | 81% (69-88%) | 77% (64-85%) | 75% (62-84%) | 75% (62-84%) | 71% (57-81%) | 63% (42-78%) |


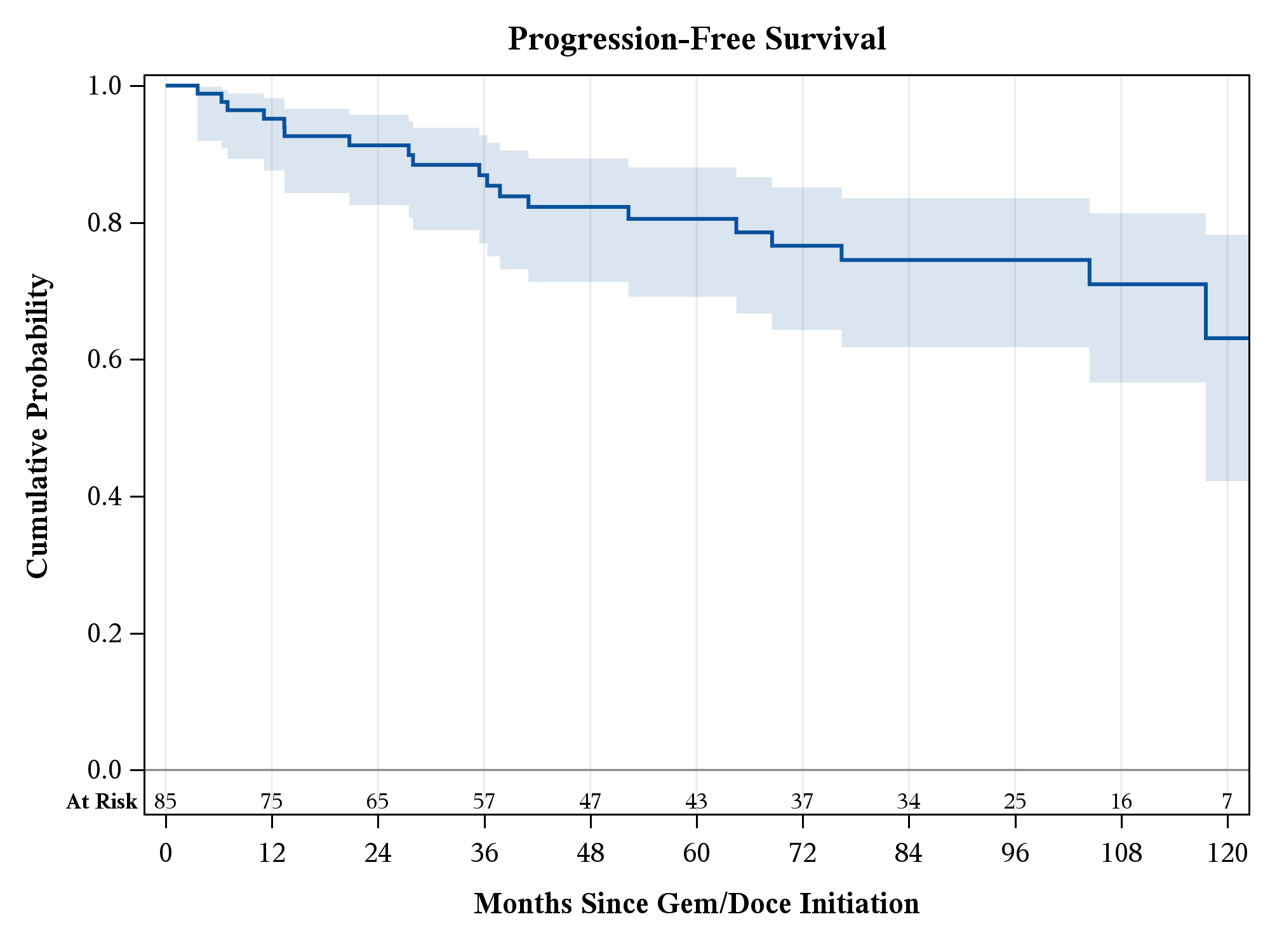


Supplementary Figure 6: Cancer-specific survival following salvage Gem/Doce

| **12 Months** | **24 Months** | **36 Months** | **48 Months** | **60 Months** | **72 Months** | **84 Months** | **96 Months** | **108 Months** | **120 Months** |
| --- | --- | --- | --- | --- | --- | --- | --- | --- | --- |
| 99% (92-100%) | 95% (88-98%) | 91% (83-96%) | 90% (81-95%) | 88% (79-94%) | 87% (76-93%) | 83% (72-90%) | 81% (69-89%) | 81% (69-89%) | 74% (55-86%) |


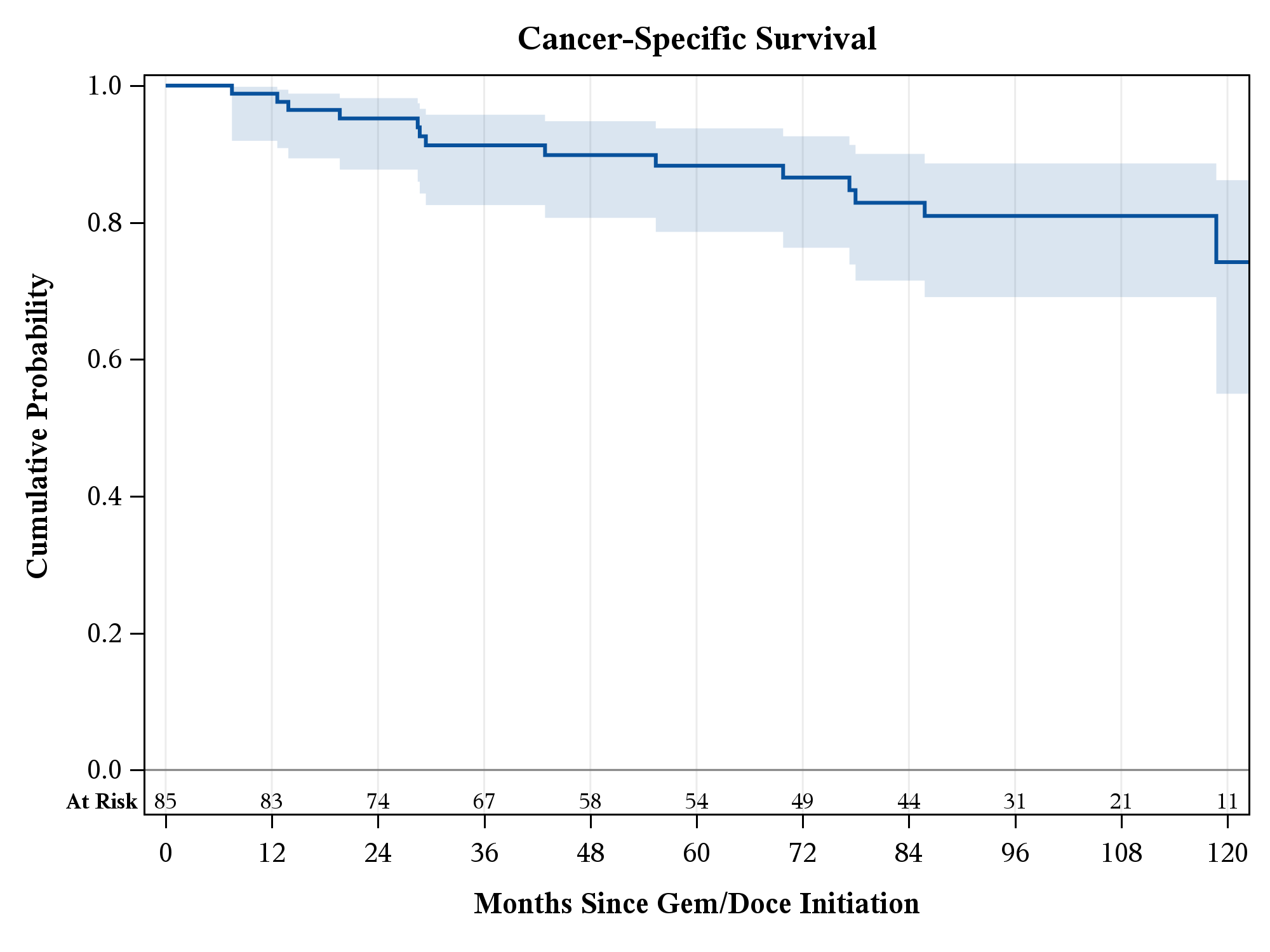


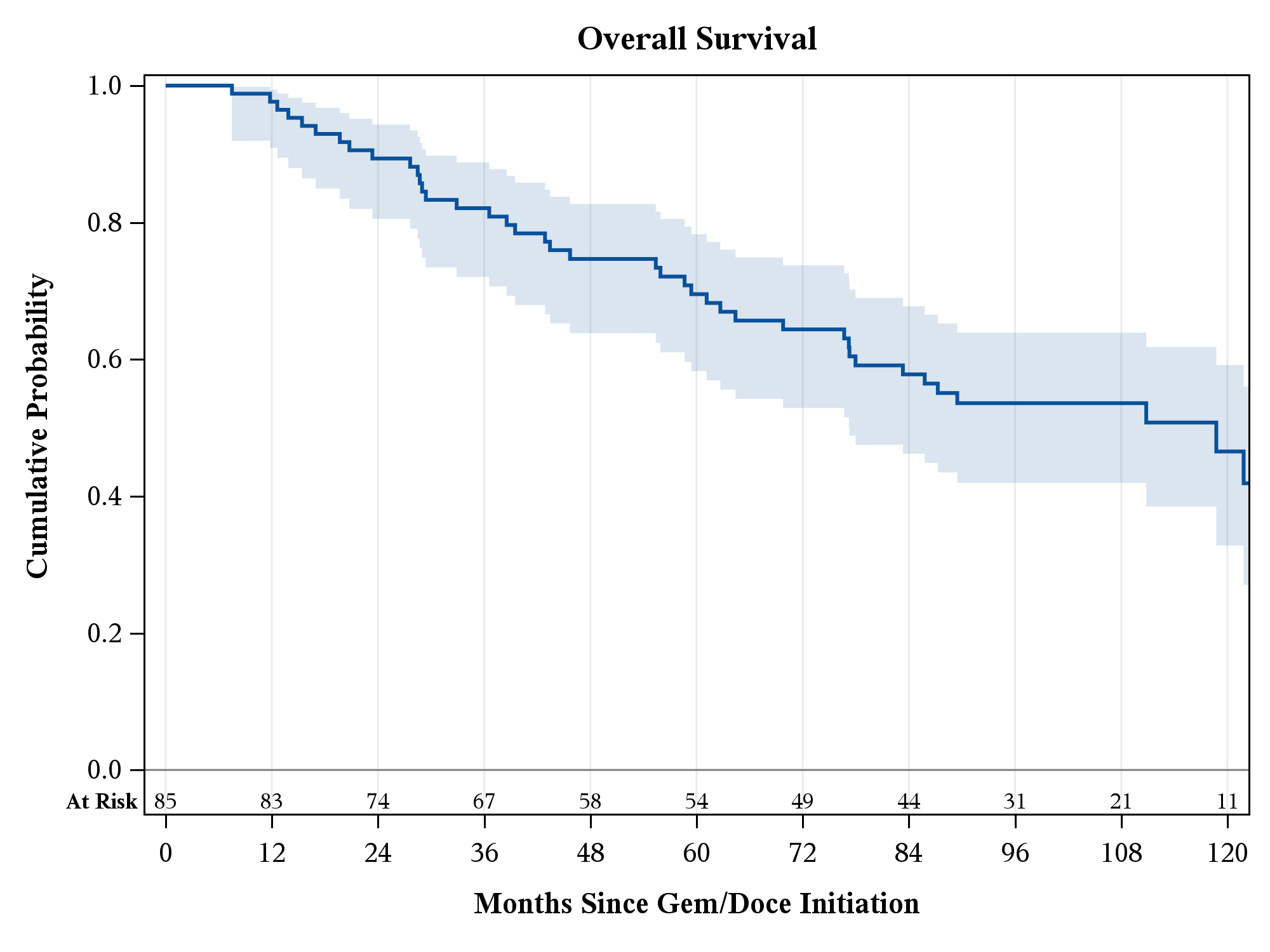
Supplementary Figure 7: Overall survival following salvage Gem/Doce

| **12 Months** | **24 Months** | **36 Months** | **48 Months** | **60 Months** | **72 Months** | **84 Months** | **96 Months** | **108 Months** | **120 Months** |
| --- | --- | --- | --- | --- | --- | --- | --- | --- | --- |
| 98% (91-99%) | 89% (81-94%) | 82% (72-89%) | 75% (64-83%) | 70% (58-78%) | 64% (53-74%) | 58% (46-68%) | 54% (42-64%) | 54% (42-64%) | 47% (33-59%) |


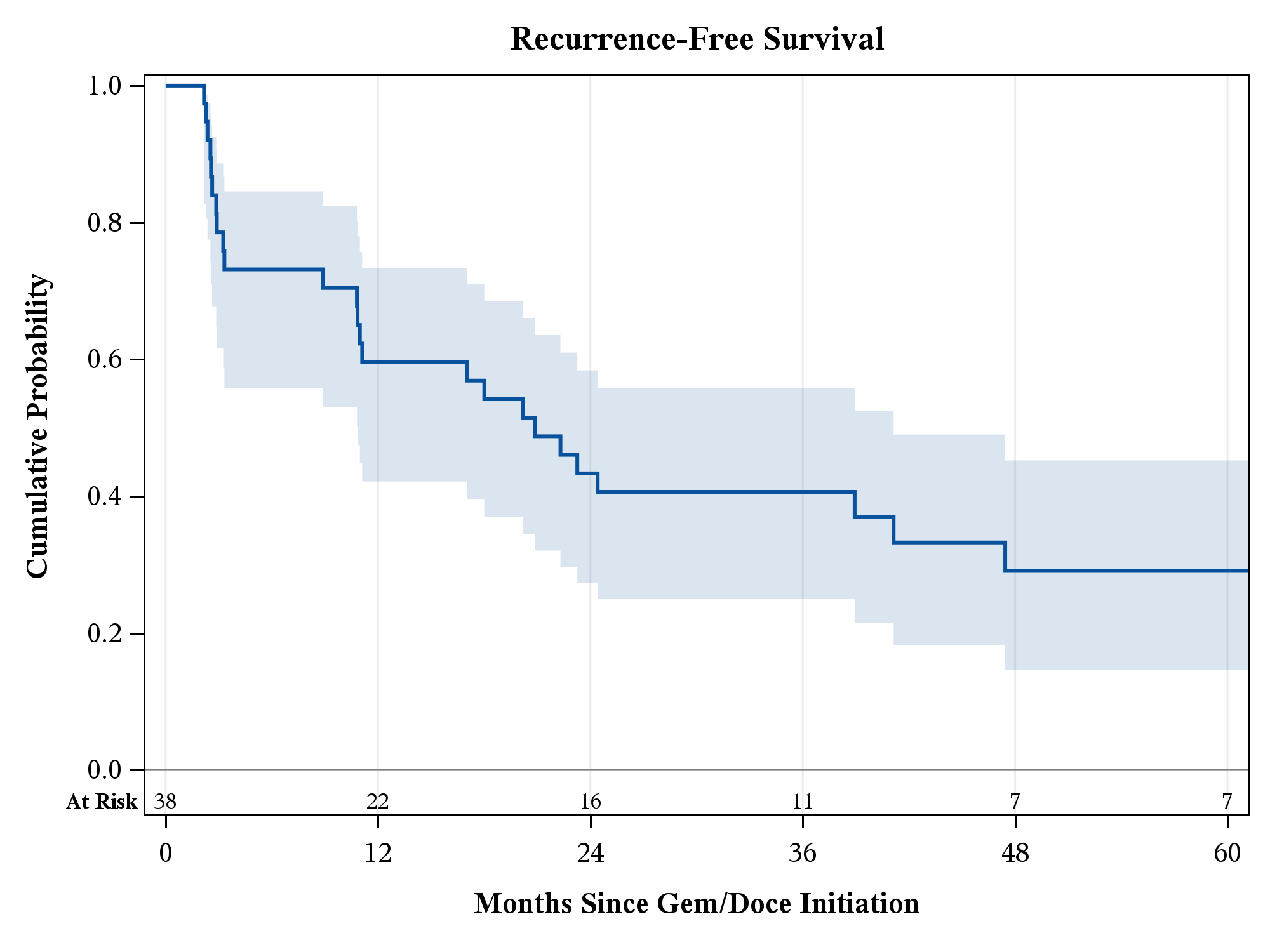
Supplementary Figure 8: Recurrence-free survival following salvage Gem/Doce among patients with BCG-unresponsive disease

|  | **12 Months** | **24 Months** | **36 Months** | **48 Months** | **60 Months** | **72 Months** | **84 Months** | **96 Months** | **108 Months** | **120 Months** |
| --- | --- | --- | --- | --- | --- | --- | --- | --- | --- | --- |
| **BCG Unresponsive** | 60% (42-73%) | 43% (27-58%) | 41% (25-56%) | 29% (15-45%) | 29% (15-45%) | 21% (8-37%) | 21% (8-37%) | 21% (8-37%) | 21% (8-37%) | 21% (8-37%) |
| **BCG Exposed** | 57% (42-70%) | 50% (35-64%) | 37% (22-51%) | 34% (20-48%) | 34% (20-48%) | 28% (15-42%) | 25% (12-39%) | 25% (12-39%) | 25% (12-39%) | 25% (12-39%) |

Supplementary Figure 9: Progression-free survival following salvage Gem/Doce among patients with BCG-unresponsive disease


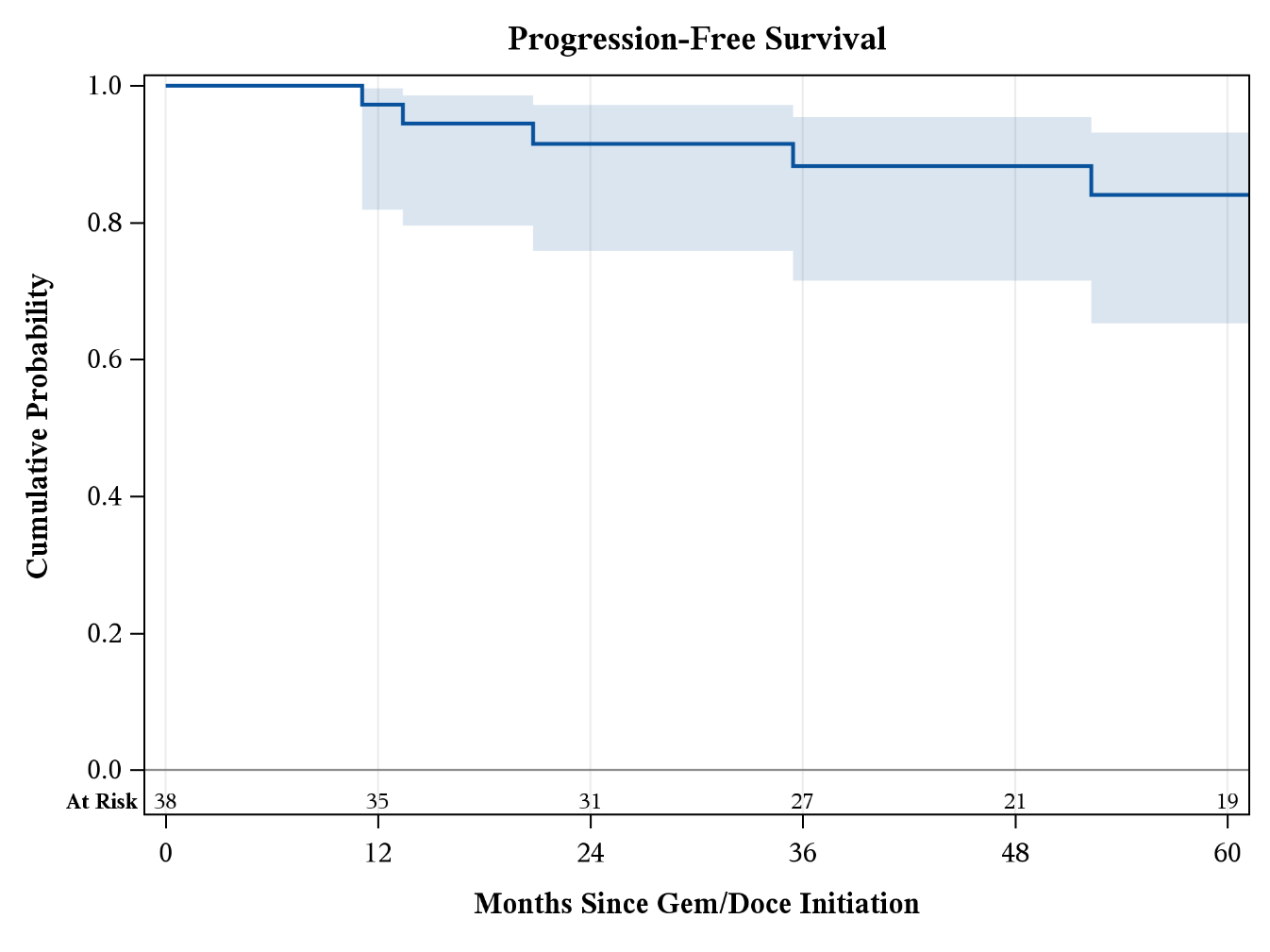


|  | **12 Months** | **24 Months** | **36 Months** | **48 Months** | **60 Months** | **72 Months** | **84 Months** | **96 Months** | **108 Months** | **120 Months** |
| --- | --- | --- | --- | --- | --- | --- | --- | --- | --- | --- |
| **BCG Unresponsive** | 97% (82-100%) | 91% (76-97%) | 88% (71-95%) | 88% (71-95%) | 84% (65-93%) | 79% (59-90%) | 79% (59-90%) | 79% (59-90%) | 79% (59-90%) | 53% (10-84%) |
| **BCG Exposed** | 93% (81-98%) | 91% (78-97%) | 86% (71-93%) | 77% (60-88%) | 77% (60-88%) | 74% (56-85%) | 70% (52-83%) | 70% (52-83%) | 64% (43-79%) | 64% (43-79%) |


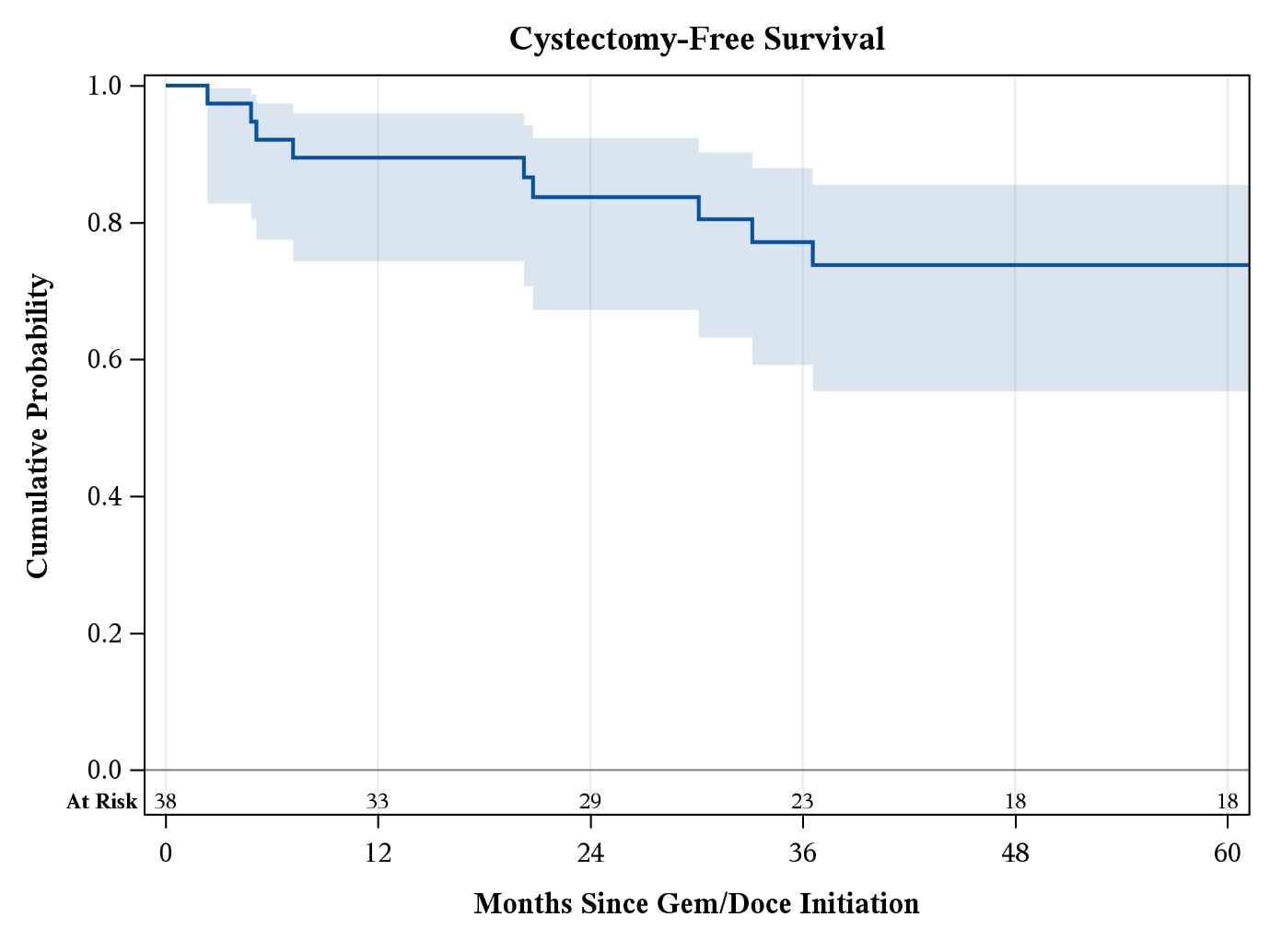
Supplementary Figure 10: Cystectomy-free survival following salvage Gem/Doce among patients with BCG-unresponsive disease

|  | **12 Months** | **24 Months** | **36 Months** | **48 Months** | **60 Months** | **72 Months** | **84 Months** | **96 Months** | **108 Months** | **120 Months** |
| --- | --- | --- | --- | --- | --- | --- | --- | --- | --- | --- |
| **BCG Unresponsive** | 89% (74-96%) | 84% (67-92%) | 77% (59-88%) | 74% (55-86%) | 74% (55-86%) | 69% (50-83%) | 69% (50-83%) | 69% (50-83%) | 60% (34-78%) | 60% (34-78%) |
| **BCG Exposed** | 87% (73-94%) | 84% (70-92%) | 84% (70-92%) | 78% (62-88%) | 75% (58-86%) | 75% (58-86%) | 67% (48-80%) | 67% (48-80%) | 60% (39-76%) | 60% (39-76%) |

Supplementary Figure 11: Metastasis-free survival following salvage Gem/Doce among patients with BCG-unresponsive disease

|  | **12 Months** | **24 Months** | **36 Months** | **48 Months** | **60 Months** | **72 Months** | **84 Months** | **96 Months** | **108 Months** | **120 Months** |
| --- | --- | --- | --- | --- | --- | --- | --- | --- | --- | --- |
| **BCG Unresponsive** | 97% (82-100%) | 94% (80-99%) | 94% (80-99%) | 94% (80-99%) | 90% (72-97%) | 85% (65-94%) | 85% (65-94%) | 85% (65-94%) | 85% (65-94%) | 57% (9-88%) |
| **BCG Exposed** | 98% (86-100%) | 96% (83-99%) | 93% (79-98%) | 87% (71-94%) | 87% (71-94%) | 87% (71-94%) | 83% (66-92%) | 83% (66-92%) | 76% (53-89%) | 65% (35-84%) |


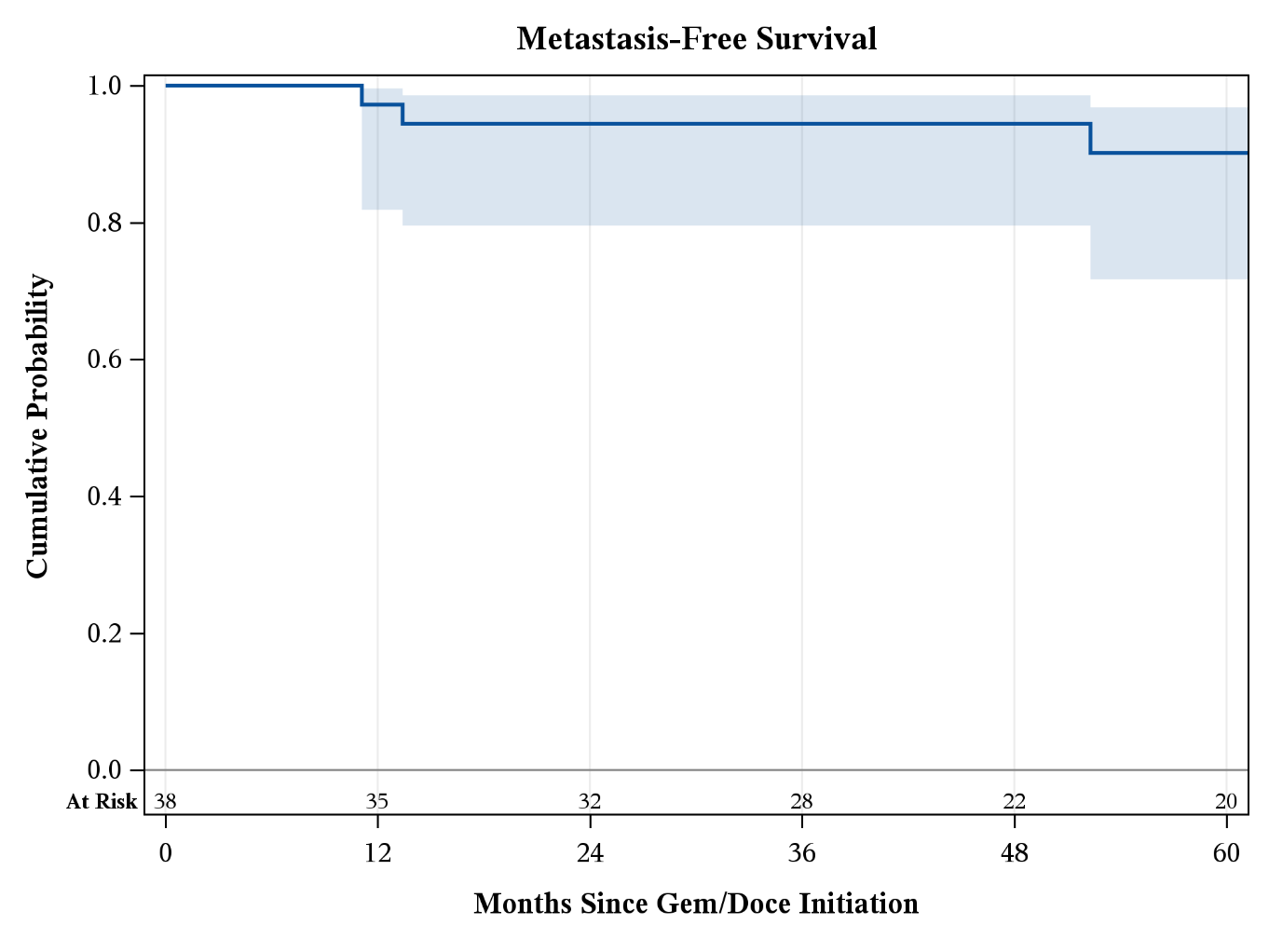


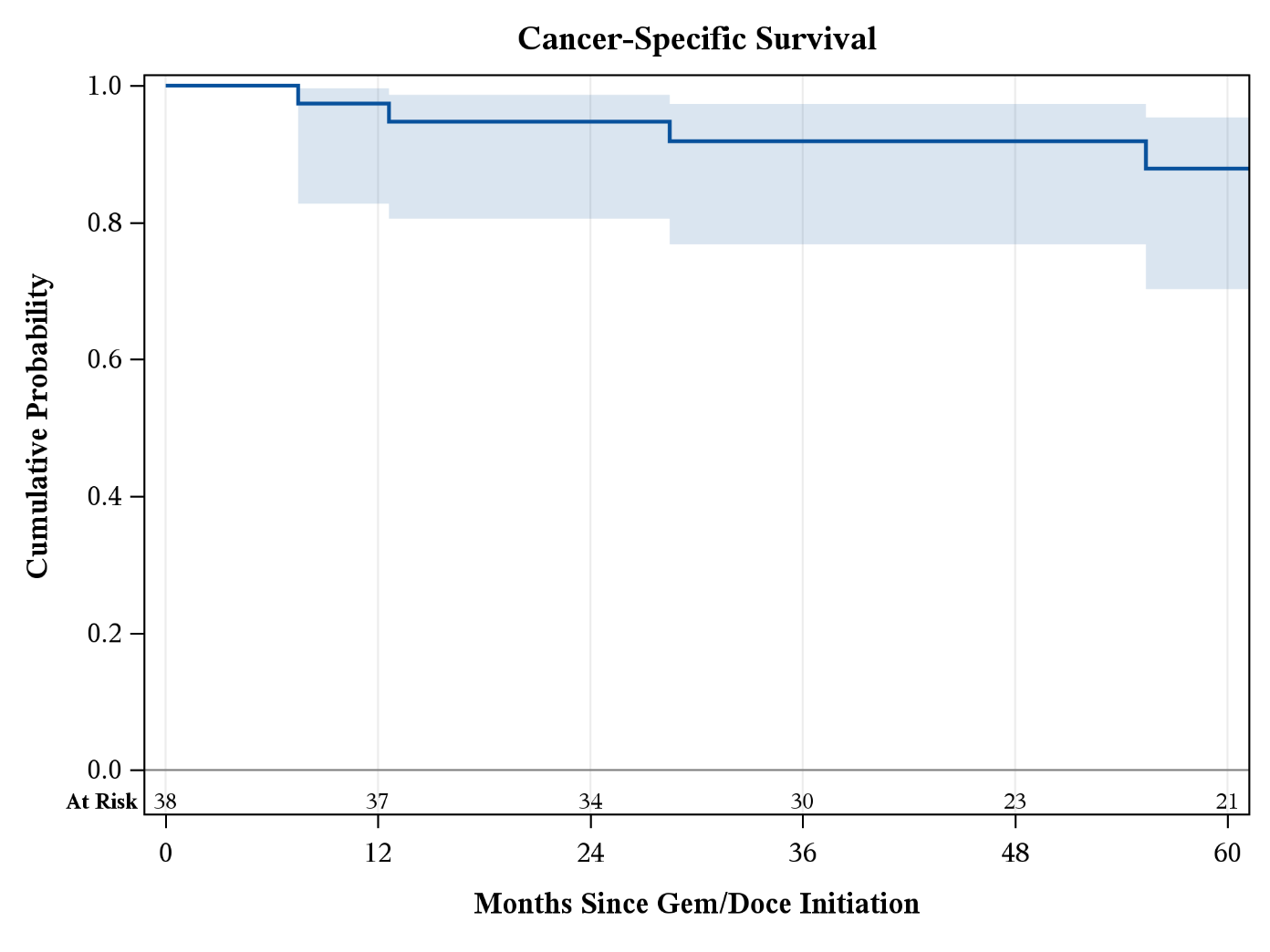
Supplementary Figure 12: Cancer-specific survival following salvage Gem/Doce among patients with BCG-unresponsive disease

|  | **12 Months** | **24 Months** | **36 Months** | **48 Months** | **60 Months** | **72 Months** | **84 Months** | **96 Months** | **108 Months** | **120 Months** |
| --- | --- | --- | --- | --- | --- | --- | --- | --- | --- | --- |
| **BCG Unresponsive** | 97% (83-100%) | 95% (81-99%) | 92% (77-97%) | 92% (77-97%) | 88% (70-95%) | 88% (70-95%) | 83% (63-93%) | 83% (63-93%) | 83% (63-93%) | 83% (63-93%) |
| **BCG Exposed** | 100% | 96% (84-99%) | 91% (77-96%) | 88% (74-95%) | 88% (74-95%) | 85% (70-93%) | 82% (66-91%) | 79% (62-89%) | 79% (62-89%) | 68% (39-85%) |


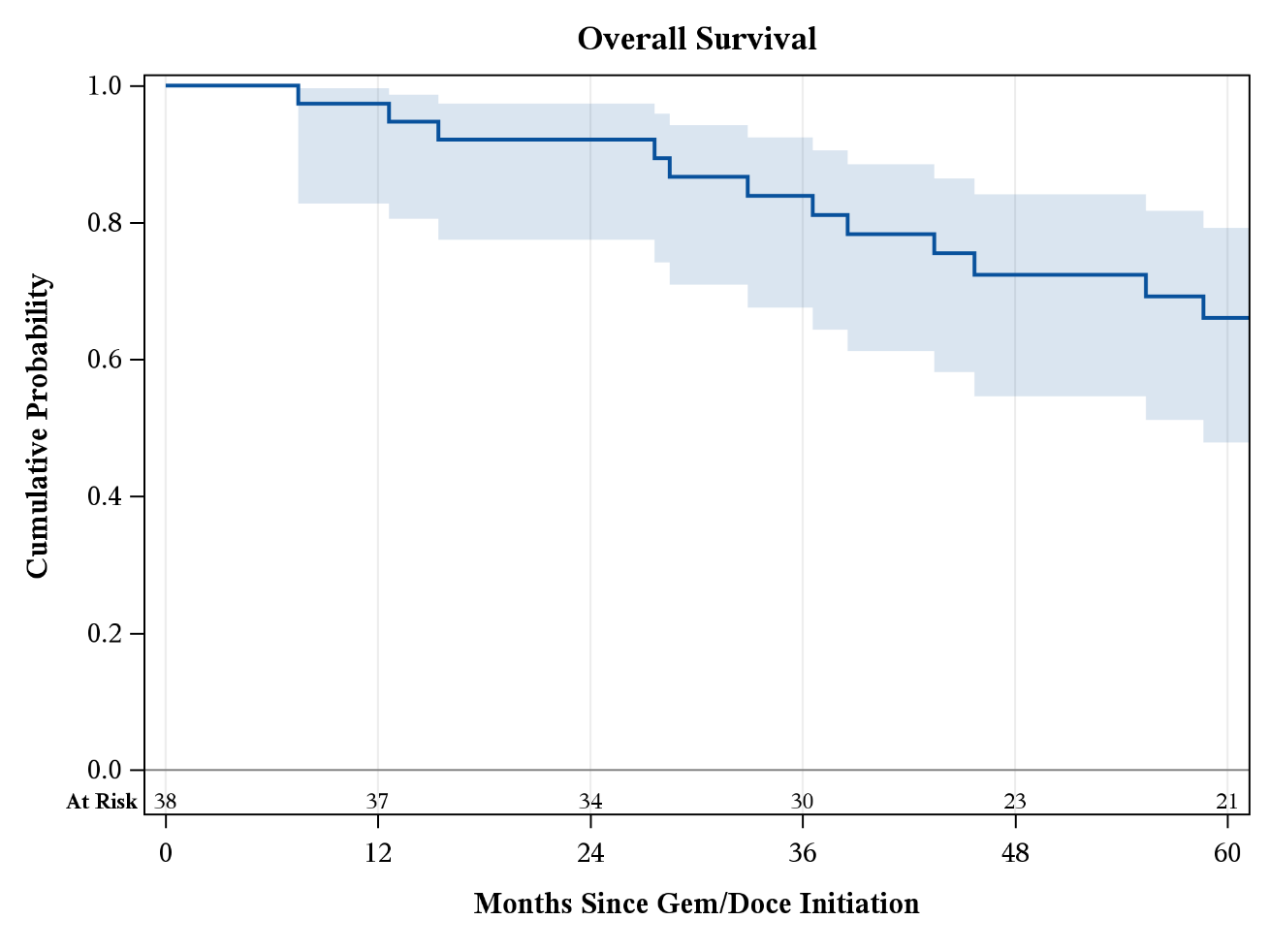
Supplementary Figure 13: Overall survival following salvage Gem/Doce among patients with BCG-unresponsive disease

|  | **12 Months** | **24 Months** | **36 Months** | **48 Months** | **60 Months** | **72 Months** | **84 Months** | **96 Months** | **108 Months** | **120 Months** |
| --- | --- | --- | --- | --- | --- | --- | --- | --- | --- | --- |
| **BCG Unresponsive** | 97% (83-100%) | 92% (77-97%) | 84% (68-92%) | 72% (55-84%) | 66% (48-79%) | 63% (45-77%) | 60% (41-74%) | 56% (37-71%) | 56% (37-71%) | 56% (37-71%) |
| **BCG Exposed** | 98% (86-100%) | 87% (74-94%) | 81% (66-89%) | 76% (61-86%) | 72% (57-83%) | 65% (50-77%) | 57% (41-69%) | 52% (37-65%) | 52% (37-65%) | 41% (23-58%) |
